# Dual Band MEMS Directional Acoustic Sensor for Near Resonance Operation

**DOI:** 10.3390/s22155635

**Published:** 2022-07-28

**Authors:** Fabio Alves, Renato Rabelo, Gamani Karunasiri

**Affiliations:** Department of Physics, Naval Postgraduate School, Monterey, CA 93943, USA; rcrabelo.br@nps.edu (R.R.); gkarunas@nps.edu (G.K.)

**Keywords:** acoustic sensor, MEMS sensor, bio-inspired, resonant sensors

## Abstract

In this paper, we report on the design and characterization of a microelectromechanical systems (MEMS) directional sensor inspired by the tympana configuration of the parasitic fly *Ormia ochracea*. The sensor is meant to be operated at resonance and act as a natural filter for the undesirable frequency bands. By means of breaking the symmetry of a pair of coupled bridged membranes, two independent bending vibrational modes can be excited. The electronic output, obtained by the transduction of the vibration to differential capacitance and then voltage through charge amplifiers, can be manipulated to tailor the frequency response of the sensor. Four different frequency characteristics were demonstrated. The sensor exhibits, at resonance, mechanical sensitivity around 6 μm/Pa and electrical sensitivity around 13 V/Pa. The noise was thoroughly characterized, and it was found that the sensor die, rather than the fundamental vibration, induces the predominant part of the noise. The computed average signal-to-noise (SNR) ratio in the pass band is about 91 dB. This result, in combination with an accurate dipole-like directional response, indicates that this type of directional sensor can be designed to exhibit high SNR and selectable frequency responses demanded by different applications.

## 1. Introduction

Directional sound sensing can be achieved with microphones that exhibit intrinsic directional characteristics or other means. The geometry of the sensor housing can be controlled to provide some directionality [1,2].

The inclusion of acoustical lenses, either conventional or more recently metamaterial-based, can provide means to conform the incoming acoustic wave [3,4,5,6]. Pressure gradient microphones achieve directionality due to a pressure gradient formed between front and back of the sensitive membranes [7,8,9]. Many directional patterns can be constructed based on designs that combine these effects [1,10]. Arrays of spatially separated microphones achieve directional sensitivity by way of monitoring arrival times and amplitudes at each microphone element. The array’s directionality is dependent on the spatial separation of the microphones and number of array elements, and directional characteristic of the individual microphones [11,12,13,14]. An alternative to microphone arrays is vector sensors. They can achieve accurate directional response relying on many different techniques and configurations and appropriate signal processing schemes [15,16,17,18,19,20,21,22]. More recently, the reduced size, lightweight, and lower power requirements of MEMS-based directional acoustic sensors fostered interest in their development [23,24,25,26,27,28,29,30,31,32,33,34,35,36,37,38]. Furthermore, the possibility of sound source localization by deploying a set of networked sensors make them even more attractive. Most of the MEMS directional sensor designs are based on the configuration of the hearing mechanism of the *Ormia ochracea* fly [23,24,25,26,27,28,29,30,31,32,33,34,35,36,37,38]. The fly’s hearing organ evolved to employ unique mechanically coupled eardrums that results in remarkable directional sound sensitivity, regardless of their small subwavelength eardrum separation [39,40].

**Figure 1:**
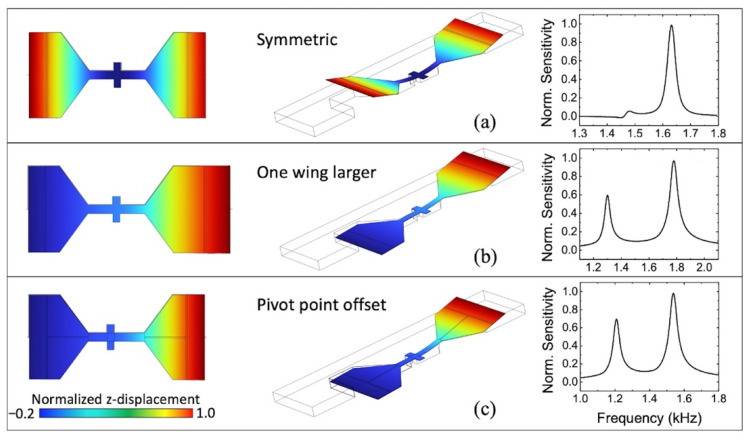
Schematic diagrams of two-wing MEMS sensors showing simulated frequency response in three different configurations: (**a**) symmetric bridge; (**b**) different wing sizes; (**c**) asymmetric leg pivot points (offset from the center). The vibration amplitudes are normalized for clarity.

A typical two-wing MEMS sensor structure designed by our group, inspired by the tympana configuration of the *Ormia ochracea*, consists of two freestanding wings coupled by a bridge, with the entire structure connected to a substrate using two torsional legs perpendicular to the bridge, as shown in Figure 1a. Sensor wings vibrate out of the substrate plane due to the sound pressure wave impinging on them, according to the oscillation modes of their structure. There are two predominant vibration modes of the mechanical structure of the sensor, namely bending and rocking modes [25], and the wings’ displacement amplitudes are a function of the incident sound frequency, sound pressure level (SPL), and direction of arrival (DOA).

It is important to highlight this sensor does not use the same mechanism of amplification of directional cues as the *Ormia ochracea*. It is rather operated with open back where the most sensitive vibration mode is the bending due to the pressure difference between top and bottom sides of the wings [32]. Thus, the sensor exhibits a cosine dependence of the wing vibration amplitudes on the angle of incidence of sound with respect to the sensor normal [32], similar to pressure gradient microphones. The rocking mode, if needed, can be made to predominate by closing the back of the sensor [35] and using asymmetric wing sizes, similar to shown in Figure 1b.

The determination of the DOA requires measurement of the sensor’s wings vibration amplitudes, which needs a transduction mechanism converting them into a more easily measurable quantity. Despite the proposition of some well-thought transduction mechanisms, stemming from photonics [24,25,26,27,28] and piezoelectric sensing [29,31], our research group chose one that could be easily incorporated into the device during its fabrication process, without the need of additional processing steps, by etching interdigitated comb finger capacitors structures at the interface between the wings’ edges and the substrate. As the wings vibrate out of the substrate plane, the overlap between the fingers attached to them and the fixed fingers attached to the substrate is altered, along with the associated capacitance between them. By utilizing capacitance-measuring circuitry, a voltage signal representative of the wing motion is obtained. The details of the MEMS sensor operation and determination of the DoA can be found in Wilmott et al. [32].

While they can be used for wide band detection when operated away from the resonance, near the resonance, a greater SNR is achievable. The reason for that is the predominance of the electronic readout noise over the mechanical noise [32] allowing for the signal to be enhanced by resonant vibration whereas the noise remains nearly the same. By tuning the sensor resonance to the desired band, high sensitivity is achievable. This narrowband characteristic is interesting for applications that require tonal detection and are not meant for audio applications that require broadband, exhibited by conventional microphones. Additionally, more than one resonant peak can be achieved by design, which can be interesting for applications requiring high SNR in a relatively broader frequency range [41,42,43]. On the other hand, by placing the resonant peaks apart, two or more acoustic bands can be detected simultaneously. The latter configuration is particularly interesting for signature-based detectors where a significant portion of the signal processing is embedded in the sensor mechanical characteristics and not in the processing electronics [44,45].

In two-wing MEMS sensors, double resonance has been achieved by designing wings with different dimensions [44,45] (Figure 1b). While increasing the area of one wing in relation to the other increases the mass and therefore decreases the frequency of resonance of that wing, it also could increase the size of the device. In this case, the different sizes of the wings make the sensor more prone to imperfections and tolerances of the fabrication process. A more convenient way to achieve double peak resonance is by making the bridge asymmetric by offsetting the pivot points of the torsional leg (Figure 1c). In this configuration, the cantilever-like bridge length is different for each wing. In a simple cantilever beam, the natural frequency is inversely proportional to the square of the length of the beam, making the leg offset the sole parameter that controls the separation of the resonant peaks. In this paper, we report on the design and characterization of a two-wing MEMS directional acoustic sensor with two separate bending resonance peaks. In this design, the phase difference between two adjacent resonances is used to increase the bandwidth of detection while maintaining high sensitivity due to the operation near resonance and using capacitive comb finger readout. Furthermore, different operations with the signal from two wings allow for the manipulation of the sensor’s frequency response.

## 2. Sensor Design

The premise for the sensor design is the narrow-band operation and the fact that the mechanical response of the individual wings can be approximated to a driven damped harmonic oscillator, whose oscillation amplitude (*A*) can be modeled as [46]:

(1) AS=F0Kω0ω(ω0ω−ωω0)2+1Q2

where *F*_0_ is the driving force (in our case the force exerted by the acoustic pressure), *K* is the spring constant, *ω*_0_ is the natural angular frequency of the oscillator, *ω* is the angular frequency of the driving force (acoustic frequency), and *Q* is the quality factor. The amplitude near resonance (*ω ≈ ω*_0_), where our sensor is intended to be operated, is approximately *A_SO_* = *Q × F*_0_/*k* whereas the amplitude away from resonance, where conventional microphones are operated, is approximately *A_SM_* = *F*_0_/*k*. The ratio between them, *A_SO_*/*A_SM_*_,_ is simply the quality factor, which is the physical limit in performance enhancement that can be achieved.

On the other hand, noise amplitude spectral density can be approximated by [23]:

(2) $$A_{N}=\sqrt{\frac{4k_{B}TQ}{\omega_{0}K}}$$

where *k_B_* is the Boltzmann constant, *T* is the temperature. Away from resonance, where the frequency response is relatively flat, the quality factor in the operation band approaches the unity (*Q* ≈ 1). Thus, the ratio between noise spectral densities, *A_NO_*/*A_NM_*, becomes square root of the quality factor. These results lead to theoretical SNR enhancement of *Q*^1/2^ just by operating the senor at resonance.

The primary contribution to damping in open back MEMS sensors using comb finger capacitors comes from the viscous damping generated by the comb fingers compared to the drag damping associated with the area of the device. Thus, by controlling the comb finger damping, the quality factor can be enhanced, and consequently, the SNR.

Acoustic sensors that operate near resonance have been demonstrated by other groups. Kang et al. [47] demonstrated an interesting sensor consisting of multiple cantilevers that resonates in multiple frequencies with separate piezoelectric readouts allowing a broad band sensing between 100 and 8000 Hz. Their individual cantilevers were trenched to provide multimode low quality factor response. The authors report that beamforming and ambient noise suppression can be achieved through signal processing. The reported SNR was 52 dB. Baumgartel et al. [41] also reported on an array of piezoelectric silicon cantilever-type diaphragm transducers each one with separate resonances to cover a range between 240 and 6500 Hz when the amplitudes are added together. They report electrical sensitivity of greater than 2.5 mV/Pa. Shkel et al. [42] developed a MEMS acoustic resonator array for detection of acoustic features in noisy environments. Their array consists of 23 paddle-shaped piezoelectric cantilevers with linearly spaced resonances between 860 and 6260 Hz. The signal from each cantilever is acquired separately and made available for signal processing. The authors report sensitivities ranging from 10.8 to 202.6 mV/Pa. Common to all these sensors is the use of piezoelectric transducers, thus, providing reduced sensitivity. The sensor we demonstrated, equipped with comb finger capacitive readout, achieved much higher sensitivity and SNR, near 13 V/Pa and near 90 dB, respectively, as it is shown in Section 3.

The design of our sensor followed three steps. First, material and fabrication processes were selected. The single-layer freestanding membrane characteristic of this type of sensor favors the SOIMUMPs process from the commercial foundry MEMSCAP, described in detail by Cohen et al. [48]. The process employs a silicon-on-insulator (SOI) substrate with 3-masking steps to define the MEMS structure. These steps establish the device layer, provide the electrical contacts, and trench the substrate. The substrate is a 400 μm thick n-type double-side polished SOI wafer with a 25 μm thick device layer separated by a 1 μm thick oxide layer. The foundry provides a set of design rules and constraints [48] that must be respected during the design to assure the device is manufacturable. Initially, 20 nm of chromium and 500 nm of gold are stacked and patterned through liftoff for the contact tracks and pads. Then, the structure of the sensor is patterned on the device layer via deep reactive ion etch (DRIE). Next, the front side of the wafer is protected, and the trenches are etched from the bottom using DRIE, stopping at the buried oxide layer. Wet oxide etch removes the oxide and front side protection is stripped using a dry etch process releasing the mechanical structures.

Second, the desired frequency response is used as a tight requirement to define the geometry of the sensor. We started with a legacy configuration from our research group [32] and estimated the geometrical parameters to obtain a single bending resonance centered around 690 Hz. Then, the symmetry is broken by offsetting the torsional beam pivot points as depicted in Figure 1c, to obtain the desired double bending characteristic.

Lastly, adjustments in the structural design and electronic readout are made to compensate effects of the fabrication, such as deformation by stress, fabrication tolerances, placement of bonding pads, electric insulation, etc.

### 2.1. Modeling and Simulation

Much work has been done in analytically modeling this type of sensor [39,49,50,51,52]. While the models are relatively accurate for specific designs and configurations, the complex nature of the structural interactions and vibrational modes makes finite element (FE) the preferable tool for accurately designing such sensors.

In our study, COMSOL Multiphysics is used where acoustic interaction with the modeled sensor structure is achieved by coupling the pressure acoustic, structural mechanics, and thermo-viscous acoustics physics’ modules. The material properties are obtained from the foundry and complemented with data from open literature and our in-house experimentation. During the simulation, the two ends of the legs away from the bridge were fixed and the entire structure was surrounded by a spherical air volume. The spherical volume was surrounded by a sound absorbing layer to eliminate the scattered sound hitting back on the sensor. Excitation of the mechanical structure is done with an acoustic wave with a chosen SPL, usually 1 Pa, so all output displacement values are provided in terms of the displacement per unit sound pressure, also known as the mechanical sensitivity (μm/Pa). For frequency response evaluation, a parametric sweep of the sound frequency is performed and displacements at the wings’ tips are recorded.

Different from conventional MEMS microphones, our sensor operates with an open back and the wings are attached to each other and to the substrate frame by small pivot points. In this configuration, there are primarily two different damping mechanisms in our sensor: (a) drag of the wings and (b) viscous damping associated with narrow air gaps of the comb finger capacitors. Through the simulations, it was verified that indeed the predominant source of damping comes from the comb fingers. Thus, damping effects in our sensor can be controlled by adjusting the air gap between fixed and moving comb fingers. Larger air gaps minimize the damping, increasing the quality factor. On the other hand, larger gaps reduce the differential capacitance as the wings vibrate in response to the incident sound, reducing the sensitivity of the sensor. The comb fingers were designed with 2.5 μm gap to obtain a quality factor slightly higher than 10.

**Figure 2:**
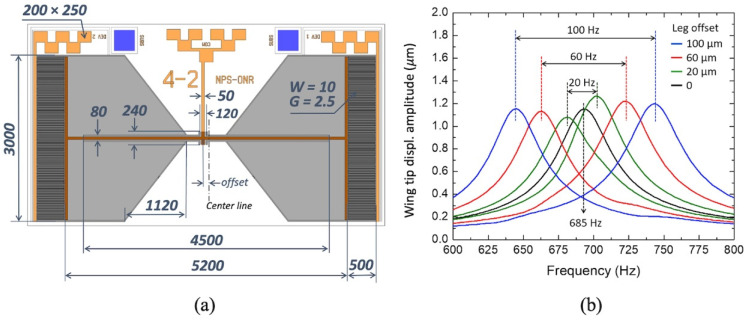
MEMS sensor design: (**a**) Schematic diagram of the design sensor with major dimensions (all dimensions are in micrometers). (**b**) Simulated mechanical sensitivity of the tip of the wings for the symmetric bridge and three different offsets. The peaks at higher frequencies are from the left wing (shorter bridge) and the peaks at lower frequencies are from the right wing (longer bridge).

The sensor depicted schematically in Figure 2a was modeled and the mechanical sensitivity was computed at the tips of the wings. Notice that in this design, the bridge is made longer by protruding through the wings to achieve the desired resonant frequency keeping the size of the sensor relatively compact. The amount of asymmetry, marked in Figure 2a as “*offset*” was increased from 0 to 120 μm resulting in a difference between bridge sizes from 0 to 240 μm. The simulated mechanical sensitivities of both wings are plotted in Figure 2b.

Note that the resonant frequency separation (∆*f*) obtained using the FE model increases 1 Hz per 1 μm of offset. This relation can be used to compute initial values for future designs where a different ∆*f* is desired.

### 2.2. Fabrication

The configuration with a 60 μm offset was selected for fabrication because it provides nearly twice the bandwidth of the symmetric configuration while preserving sensitivity. Larger offsets will provide broader responses with the expense of reducing sensitivity in between the two peaks while and increasing the noise bandwidth as observed by the reviewer. A first order approximation shows that at the resonant peaks the resulting SNR due to the offset will be reduced by the ratio between the bandwidths with and without the offsets.

**Figure 3:**
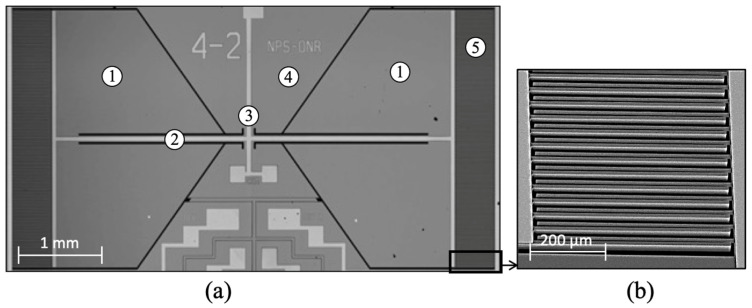
Fabricated MEMS sensor: (**a**) Optical microscopy image of the Gen 4-2 sensor. Metallized pads and tracks appear brighter than the substrate gray. The numbers on the image represent: ① Wing; ② Bridge; ③ Pivot point; ④ Substrate; ⑤ Capacitive comb fingers. (**b**) Scanning electron microscope image of the comb finger capacitors.

The comb finger capacitors were placed at the edges of the wings, which exhibits the largest displacement. The fabricated sensor was named generation 4-2 (Gen 4-2), shown on the micrograph in Figure 3a. The light gray lines and polygons are metallic lines and contact pads. Figure 3b shows a closeup of the comb finger capacitors.

A vertical misalignment between moving (wing) and static (substrate) comb fingers is noticeable in Figure 3b. It is known that the SOIMUMPs process introduces residual stress to MEMS structures due to the fabrication process [48,52,53]. At the end of the process, when the sensor structure is released from the substrate, the residual stress warps the wings upward, thereby displacing their equilibrium position at rest and decreasing comb-fingers overlap [52]. If this displacement becomes large, it may reach a condition of small to no overlap between comb fingers, resulting in substantial reduction of the static capacitance [52]. In the SOIMUMPs process, the stress gradient in the device layer used for creating the mechanical structures originates primarily during the doping step [48]. In addition, the difference in coefficients of thermal expansion is also significant when multiple layers are in contact [54,55]. This effect can be incorporated in the model if the stresses of the constituent layers are known.

The electro-mechanical transduction is achieved using a charge amplifier that translates differential capacitance of the comb fingers due to vibration of the wings to a voltage proportional to the displacement of the wing tips. Stages of gain and filters can be added to appropriately condition the signal to the intended application. In this configuration, to maximize the electro-mechanical transduction (V/μm) and, consequently, the electric sensitivity of the sensor (V/Pa), it is necessary to make sure there is an overlap between moving and static comb fingers when the sensor is at rest [52]. This overlap must be greater than the maximum displacement caused by the acoustic stimulus to minimize non-linear effects.

Finally, the torque induced by gravity in a first order approximation is about 8 μNm (wing mass, *m* ≈ 260 μg and wing length is about 3 mm) at the tip of the wing. If the sensor is perpendicular to the force of gravity (horizontal), the wings should move downwards about 4 μm (*x* = *g*/*ω_0_*^2^), which is less than the observed upward curling of the wing of about 15 μm due to residual stress. Our sensor is operated on a vertical position (parallel to the force of gravity) to measure azimuth. On that position, the change in stiffness due to the pivoting characteristics significantly reduces the downward displacement, which is slightly less than 1 μm. During characterization and operation, no arcing or shorting in the comb fingers, whose gap is 2.5 μm, was noticed.

## 3. Results and Discussion

### 3.1. Sensitivity

**Figure 4:**
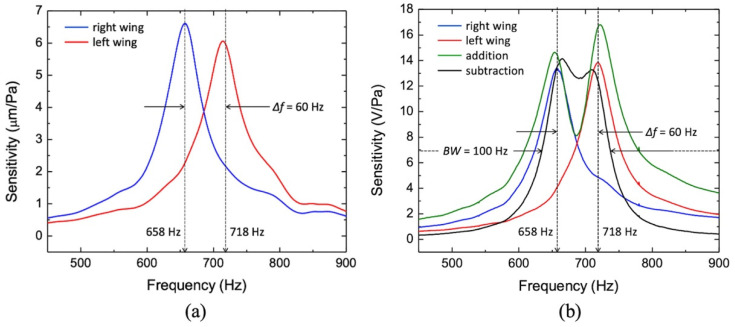
Sensitivity measurements: (**a**) Mechanical sensitivity measured using laser vibrometry at each wing tip separately. (**b**) Electric sensitivity of both wings measured using lock-in amplifiers (blue and red lines). Addition and subtraction of the wings output signals are also shown (black and green line, respectively).

Initially, the sensors were submitted to a known acoustic excitation in an anechoic chamber while monitoring their wings’ mechanical displacements. Figure 4a shows the measured mechanical sensitivities for each wing, which are over 6 μm/Pa at resonance for both wings. It can be observed that the measured frequency responses are in close agreement with the corresponding simulations.

The amplitude differences observed when comparing with simulation and measurement may be attributed to the model not including all the structures surrounding the sensor in the actual measurement setup (sensor mount, optical bread board, motion stages, vibrometer head, etc.). These structures can act as baffles, which can disturb the pressure field around the sensor and the displacement amplitude since the sensor operates in pressure gradient mode [32]. As discussed previously, a mechanical to electrical transduction mechanism is needed for practical application of the sensor. For the sensors presented in this work, the capacitance change induced on the comb finger capacitors due to the wing’s displacement (pF/μm) is further converted into a voltage by a capacitance measuring circuit (V/pF). The sensitivity is then referred as an electrical sensitivity (V/Pa).

For the next set of measurements, the sensor was attached and wire-bonded to a printed circuit board (PCB) containing a charge amplifier, additional gain stages, and Sallen–Key filters. A hole was drilled on the PCB right underneath the sensor to expose its back side. The electronic readout is AC coupled to filter out DC and low frequencies. The advantage of this scheme is that since changes in temperature and humidity due to the environment are slow processes compared with the vibration of the wings, they do not interfere with the voltage output response. This electrical readout was not optimized for low noise and is rather intended to show the potential of this sensor configuration in terms of multi-band responses. Figure 4b shows the electrical sensitivity curves (red and blue solid lines) of each wing, measured separately, which, for both wings are over 13 V/Pa at resonance. This corresponds to 22 dBV/Pa.

Observing Figure 4b, it is possible to notice that the electrical sensitivity associated with the wing with higher resonant frequency (red curve) is larger than the one associated with the lower resonant frequency (blue curve). This is opposite to the mechanical sensitivity plots in Figure 4a, hinting a larger electro-mechanical transduction for the wing with the shorter bridge and it can be attributed to a non-uniform frequency response of the amplification stage.

**Figure 5:**
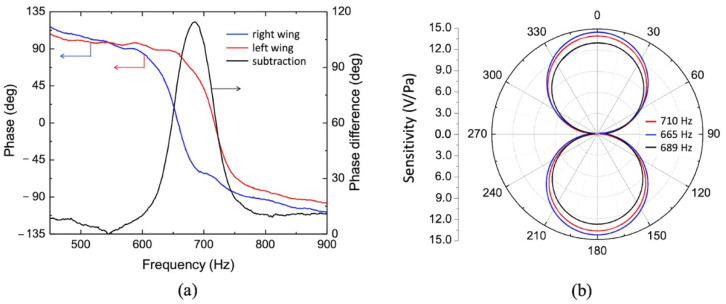
Phase and directionality characterization: (**a**) Measured phase frequency response for both wings separately (left side scale) and phase difference (right side scale). (**b**) Directional response measured at the peaks and valley between peaks of the subtraction of the two wing output signals, showing in Figure 4b, solid black line.

This particular sensor configuration exhibits an advantage of allowing for modifying the frequency response. To obtain a single broader band or two narrower separate bands, the response of both wings were electronically subtracted and added, respectively. This can be seen in Figure 4b (black and green solid lines). This is possible due to the phase inversion at the resonances. Figure 5a shows the measured phase response of the signal generated by both wings (red and blue lines) as well as the phase difference (black line). It can be seen in Figure 5a that the two phases are separated by nearly 180 degrees between the two resonance peaks making the subtraction equivalent to adding of two signals as illustrated in black line of Figure 4b. Thus, it is possible to generate four different responses by properly selecting the outputs of the electronic readout.

### 3.2. Directionality

Finally, Figure 5b shows the directional response of the sensor, measured at peaks and valley between peaks of the subtraction of the two wing output signals (Figure 4b, solid black line). For this measurement, the sensor assembly was mounted to a turntable inside the anechoic chamber and the specific tones were played one at a time while a full azimuth rotation at 1°/s rate was executed by the turntable. At all frequencies, a dipole-like directional response was obtained where the maxima were obtained for normal incidence and 180°. By fitting a cosine curve to the normalized directional response (not showing in Figure 5a, the cosine dependence on the angle of incidence was confirmed. An accurate method to evaluate the cosine directional response through 3 dB polar width was demonstrated by Wang et al. [56]. This directional behavior is expected since the sensor is operated with open back and the bending vibrational mode is dependent on the pressure gradient (∆*P*) between front and back. Assuming the presence of the sensor in the acoustic field does not affect the pressure gradient (dimensions are much smaller than the wavelength), for an incident plane wave the force exerted in the sensor’s wings (*f*(*t*)) is a composition of the front side (*p*(*t*)) and the diffracted, time-delayed back side acoustic pressure (*p*(*t* + *τ*)). A very simplified mathematical representation can be given by:

(3) f(t)=A[p(t)−p(t+τ)]

where *A* is the area of the wing and *τ* is the time it takes for the wave to be transmitted around the sensor and baffle, to the back and it can be approximated by [57] *τ* = (*L*/*c*)cos(*θ*). Here, *L* represents the effective distance traversed by the diffracted wave, *c* is the speed of sound and *θ* is the angle of incidence. A plane wave with amplitude *P*_0_ can be represented as *p*(*t*) = *P*_0_*e^iωt^*, thus the force can be expressed as:

(4) f(t)=AP0[1−ei(2πLλ)cos(θ)]eiωt

where *λ* is the wavelength of the incoming sound. Expanding (3) since *L* is much smaller than the wavelength of sound (i.e., *L*/*λ* << 1), the force can be simplified as:

(5) f(t)≈[AP0(2πLλ)cos(θ)]

The bending motion displacement at the tip of the wing is proportional to the ratio from the force *f*(*t*) by the dynamic stiffness (displacement impedance) [57], and therefore proportional to the cosine of the angle of incidence, as it can be seeing in Figure 5b.

### 3.3. Noise and Signal-to-Noise Ratio

**Figure 6:**
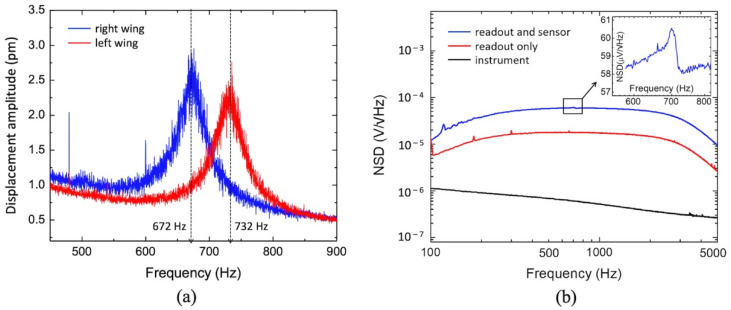
Mechanical and electrical noise: (**a**) Measured intrinsic bending vibration of the sensor in absence of any sound stimulus. (**b**) Measured electrical noise spectral density of the instrument, readout electronics with a 22 pF fixed capacitor connected to the input of the charge amplifier (readout only) and readout electronics with the MEMS sensor connected. The inset shows a zoom in of the area highlighted by the square, where the vertical scale is linear for better view of the effect.

To access the noise associated with the sensor, two experiments were performed. First, the natural vibration of the tip of both wings was measured using a laser vibrometer without any sound stimulus to access the thermomechanical noise. Figure 6a shows the resulting displacement amplitudes as a function of frequency for the two wings.

Note that the intrinsic displacement amplitude is on the order of units of picometers, and the two resonances are clearly shown. There is a 14 Hz blue shift on both resonant peaks that is most likely due to the superimposition of the 1/f noise on the sensor response in addition to stress impinged on the sensor by the mount. At resonance, for typical MEMS devices, a first order estimation of the intrinsic noise equivalent deflection (NED), originated from thermal noise and computed in m/√Hz, can be done by slightly modifying Equation (2) to the following expression [23,56,58]:

(6) $$NED_{th}=\sqrt{\frac{4k_{B}TQ}{m{\left(2\pi f_{0}\right)}^{3}}}$$

where *m* is the mass of the wing and *f*_0_ is the frequency of resonance. Using (4) with *T* = 290 K (anechoic chamber temperature), *Q* = 11 (extracted from the data showing in Figure 5a), *m* ≈ 260 μg (estimated mass of the longer bridge plus its attached wing), and *f_0_* = 672 Hz, a *NED_th_* of 3 pm/√Hz was obtained. This is in very close agreement with the measured data showing in Figure 6a.

Equation (6) shows that the NED is inversely proportional to the square root of the mass of the membrane, which is directly related to size (area). Unfortunately, comparison with commercial MEMS microphones is difficult since the datasheets do not include details of their membranes. Je et al. [59] report on measurements of the thermomechanical noise in terms of noise equivalent displacement, similar to what is shown in Figure 6a. In that case, displacements were measured for the sensitive membrane alone and sensitive membrane attached to a backplate. The sensitive membrane has an area of 0.2 mm^2^ and the recorded peak displacement was 380 pm at resonance. With the backplate, the mass of the device was increased approximately by a factor of 2.5 and the recorded peak displacement was 180 pm. In our case, the area of a wing is about 4.9 mm^2^ (relatively large device to obtain low resonant frequency) and the measured peak displacement was 3 pm. While it is difficult to quantitatively compare devices with very different configurations, geometry, and materials, the trend is verifiable.

Second, the noise characteristics of the sensor/circuitry and instrumentation were measured in three different conditions and the results are shown in Figure 6b. Initially, the intrinsic noise of the instrument was measured (Figure 6b black trace). Next, the influence of the capacitive readout circuit was measured. Ideally, this should be done with an unreleased sensor; however, the foundry does not offer such option. Alternatively, the capacitance of the sensor was measured using a parametric analyzer to be around 22 pF (2 pF from the comb fingers and 20 pF from the die) and a fixed capacitor of the same value was connected to the inputs of the charge amplifier (Figure 6b red trace). The sharp peaks around 200, 300, and 700 Hz are most likely to be noise spikes, related to adjacent laboratory activities and uncorrelated with the circuit response. Then, the readout circuit with the MEMS sensor connected was measured in complete dark to verify the influence of the thermal and shot noise, and stray capacitances from the sensor die as well as any intrinsic vibration that could be possibly transduced (Figure 6b blue trace). The scales in Figure 6b are logarithmic to better show the noise characteristics. The instrument noise was measured unfiltered and shows the characteristics of 1/f noise with logarithmic decaying with frequency. Since the sensor is meant to operate near resonance, the readout circuit has a bandpass filtering stage with cutoff frequencies around 150 and 3000 Hz. The filter characteristic is clearly observable in the curves “readout only” and “readout with the sensor”, shown in Figure 6b. It is noticeable that the noise measured with the fixed capacitor replacing the sensor is about 3.5 times smaller than the noise measured with the sensor. This difference may be attributed to the noise generated in the doped silicon die. No resonant behavior in this range of frequencies is shown, except for a minute blip seen around 700 Hz where the mechanical vibration noise is predominant (inset of Figure 6b). Taking the resonant peak of the left wing, which is closer to 700 Hz (718 Hz) the ratio between the electrical sensitivity (13.7 V/Pa) and the mechanical sensitivity (6 μm/Pa) is 2.45 V/ μm. A mechanical vibration of 2.5 pm (Figure 6a) causes an output voltage of 61 μV, which is very close to the measurement shown in the inset of Figure 6b. The right-wing mechanical vibration at resonance causes an output voltage of 56 μV, which is below the overall noise.

This is an indication that the electronic noise due to the sensor’s die is predominant and the mechanical noise does not show a significant contribution. Further studies must be conducted to evaluate the impact of the size of the sensor die in the overall noise. For an incoming sound pressure of one pascal, the signal-to-noise ratio (SNR) in the passband, 120 Hz wide, can be as high as 91 dB.

Comparison between sensors intended for different applications (e.g., conventional MEMS microphones) is somewhat difficult; however, the following comparison is intended to demonstrate that the same SNR levels could not be achieved by merely limiting the bandwidth of commercial MEMS microphones, as predicted in Section 2. The narrowband acoustic sensor demonstrated here (bandwidth shorter than 1/3 octave band around the passband) operates near 1 kHz. A-weighing does not change much the noise of the sensor and the resulting SNR is approximately 92 dBA.

Another important point is that in most commercial MEMS microphones, the MEMS device is attached to a dedicated ASIC, which provides the electronic transduction of the mechanical vibration. The state-of-the-art ASICs exhibit extremely low noise making the mechanical noise from the MEMS devices (self-noise) to predominate. As mentioned in Section 3.1, the readout electronics, used in our sensor, is a non-optimized prototype designed to allow testing the concept of the MEMS operation. In this case, the electronic readout noise predominates leaving much room for improvement. Nevertheless, even though a comparison of performance significantly favors the commercial devices, a few state-of-the-art commercial MEMS microphones with analog output were selected to be compared. For that, the SNR of the commercial microphones were adjusted for a 120 Hz bandwidth (same band used to calculate the SNR of our sensor, between 625 and 745 Hz) as if a noiseless narrowband filter was added to the output of the microphones. Most of the microphones were found to exhibit similar NSD, which averages about −140 dB (V^2^/Hz) within the bandwidth of our device [60,61], thus, the non-A-weighted SNR was calculated over 120 Hz bandwidth, using the sensitivity provided in the datasheets. Table 1 shows the calculated parameters. It can be seeing that our acoustic sensor exhibits a higher SNR than that of the conventional MEMS microphones over the same bandwidth.

It is noticeable that for narrow band operation, the proposed sensor, even with non-optimized readout electronics, is still a better option than the state-of-the-art conventional MEMS microphones.

In summary, these results indicate that this type of sensor can be designed to exhibit high SNR and selectable frequency responses as the applications demand. An array of such sensors could cover a much broader range of frequency bands and potentially allow for spectral analysis of several low intensity sound sources.

## 4. Methods

### 4.1. Mechanical Sensitivity Measurements

**Figure 7:**
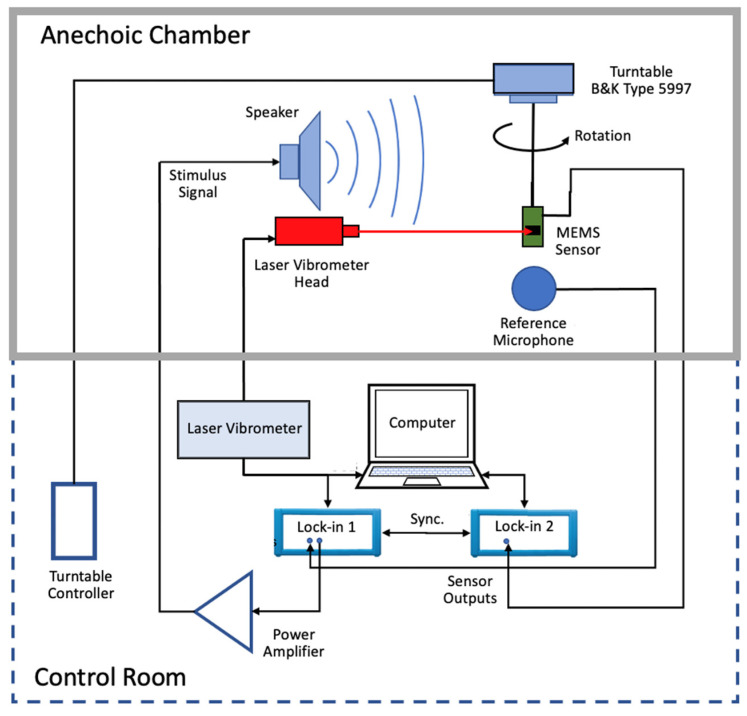
Schematic diagram of the experimental setups used to perform measurements of mechanical sensitivity, electric sensitivity, and noise. Details in the text.

For this measurement, an OFV-5000 laser vibrometer (Polytec, GmbH, Waldbronn, Germany) was utilized. The sensor was assembled on printed-circuit board (PCB) which acted as a sample holder on the experimental setup. No electrical connections were made between the sensor die and the PCB pads at this time. The vibrometer laser beam was positioned at the tip of the sensor wings. A calibrated omnidirectional microphone, model 378A21 from PCB Piezotronics, was positioned side-by-side with the sensor board, approximately 5 cm from the center of the board. The microphone calibration factor is input to the vibrometer controller in order to ascertain the SPL impinging on the sensor, assuming the sound field is not significantly different from the one on the close-by microphone. A speaker is also placed inside the anechoic chamber, about 4 m from the sensor with its longitudinal axis aligned with the mount, setting a normal incidence for the acoustic wave impinging on the sensor. A sinusoidal acoustic signal swept from 450 Hz up to 900 Hz was generated by the vibrometer, amplified, and sent to the speaker. The displacement readings for ten frequency sweep scans were averaged, resulting in the final displacement reading normalized with respect to the incident sound pressure (μm/Pa). Figure 7 shows a schematic diagram of the described experimental setup. The anechoic chamber is a 12” concrete-walled room, mechanically and acoustically isolated from the building. Absorption of 99% of the incident sound in the internal chamber is provided by fiberglass wedges (40”) for frequencies greater than 100 Hz. The anechoic chamber volume is 27′ × 14′ × 11′.

### 4.2. Electric Sensitivity Measurements

The sensor, cemented on the printed circuit board containing the electronic readout was attached to a holder and placed inside the anechoic chamber facing the speaker in a way that the incident sound wave was normal to the sensor die surface. A MLFI 500 kHz/5 MHz, 60 MSa/s (Zurich Instruments, Zurich, Switzerland) lock-in amplifier was used to measure the voltage amplitude as well as phase generated by the circuitry on the PCB at the excitation frequencies. The lock-in oscillator output synchronized to its internal reference was applied to the speaker after being amplified using the same sound amplifier as before. The calibrated microphone was once again placed near the directional sensor in order to measure the actual SPL impinging on the sensor. The microphone signal amplitude was measured by a second identical lock-in amplifier, synchronized to the first one. A frequency sweep from 450 Hz to 900 Hz was executed by a control software on a computer connected to both lock-in amplifiers. Making use of the microphone calibration factor, the voltages detected at each frequency by the second lock-in resulted in the actual sound pressure applied at each frequency of the sweep. The voltage amplitudes from the sensor, measured by the first lock-in were then divided, point-by-point, by the obtained pressure at each frequency, resulting the electrical sensitivity of the wing. Figure 7 shows the setup arrangements and connections.

### 4.3. Noise Measurements

The thermomechanical noise was performed with the same experimental setup used for sensitivity measurements, described in Section 4.2. A frequency sweep was set in the laser vibrometer; however, the output to the speaker was removed to assure the measurements were done without any acoustic stimulus. A total of 600 scans over a 2 kHz bandwidth with 12,800 frequency bins (156.25 mHz resolution) were averaged to obtain the responses shown in Figure 6a.

The same experimental setup described in Section 4.1 (Figure 7) was used to measure noise spectral densities. Initially, the lock-in input was terminated with matched 50 ohms load in order to evaluate the intrinsic noise of the instrument. The lock-in band was opened to 20 kHz and 10,000 subsequent NSD measurements with a 1.63 Hz frequency resolution were averaged (trace averaging), significantly reducing the standard deviation around the NSD average value at each frequency, resulting in a more representative noise floor measurement. The same settings were used to measure the noise of the readout electronics and sensor as described in Section 3.

## Figures and Tables

**Table 1:** Sensitivity and signal-to-noise ratio of state-of-the-art commercial MEMS microphones with analog output, including the sensor reported in this paper, for comparison.

Microphones	SNR Frequency Range (Hz)	Sensitivity (dBV/Pa at 1 kHz)	SNR for Full BW (dBA)	SNR for 120 Hz BW (dB)
Two-wing Sensor	625–745	22 (at 690 Hz)	92	91
CUI devices [62]	20–10,000	−37	62	82
IvenSense [60]	25–20,000	−36	74	83
Knowles [63]	13–13,000	−34	70	85
TDK [61]	80–20,000	−38	66	81
Infineon [64]	80–20,000	−38	73	81

## Data Availability

The data that support the findings of this study are available from the corresponding author upon reasonable request.
